# Hidden hemoplasma species within the “*Candidatus* Mycoplasma haemominutum” lineage in Thai cats revealed by analyses of two independent genetic markers

**DOI:** 10.1186/s13071-025-07112-3

**Published:** 2025-11-19

**Authors:** Kritsada Thongmeesee, Thuong Thi Huyen Bui, Duriyang Narapakdeesakul, Patchana Kamkong, Suchansa Thanee, Aung Aung, Chalida Sri-in, Wittawat Wechtaisong, Saruda Tiwananthagorn, Vito Colella, Sonthaya Tiawsirisup

**Affiliations:** 1 Center of Excellence in Animal Vector-Borne Diseases, Veterinary Parasitology Unit, Department of Veterinary Pathology, Faculty of Veterinary Science , Chulalongkorn University, Bangkok, 10330 Thailand; 2 The International Graduate Program of Veterinary Science and Technology, Faculty of Veterinary Science, Chulalongkorn University, Bangkok, 10330 Thailand; 3 Veterinary Parasitology Unit, Department of Veterinary Pathology, Faculty of Veterinary Science, Chulalongkorn University, Bangkok, 10330 Thailand; 4 Academic Service Division, National Laboratory Animal Center, Mahidol University, Nakhon Pathom, 73170 Thailand; 5 Faculty of Veterinary Medicine, Chiang Mai University, Chiang Mai, 50100 Thailand; 6 Department of Veterinary Biosciences, Melbourne Veterinary School, University of Melbourne, Parkville, VIC 3050 Australia

**Keywords:** Cat, *Candidatus* Mycoplasma haemominutum, *Candidatus* Mycoplasma *turicensis*, *Mycoplasma haemofelis*, Thailand, 16S rRNA gene, 23S rRNA gene

## Abstract

**Background:**

Hemotropic *Mycoplasma* spp. (hemoplasmas) parasitize erythrocytes and cause hemolytic anemia in several mammalian species, including cats. *Mycoplasma haemofelis* (*Mhf*), “*Candidatus* Mycoplasma haemominutum” (*C*Mhm) and “*Candidatus* Mycoplasma turicensis” (*C*Mt) are the three main feline hemoplasma species. A species closely related to *C*Mhm was recently proposed as a putative novel species based on the 23S ribosomal RNA (rRNA) gene.

**Methods:**

In this study, 16S and 23S rRNA genes were used to investigate hemoplasma diversity in cats. Blood samples from 388 cats were obtained and screened for hemoplasma infection based on a PCR assay targeting the 16S rRNA gene. Positive samples were sequenced based on the 16S and 23S rRNA genes. All obtained sequences were analyzed by the nucleotide Basic Local Alignment Search Tool (BLASTn), the DnaSP6 computer program, phylogenetic construction, genetic network and pairwise identity matrix.

**Results:**

The 388 blood samples collected from the cats were screened for hemoplasma infection. The tests showed that 68 cats (17.5%, 95% confidence interval [CI] 13.9–21.7%) were positive for hemoplasmas. Of these 68 positive samples, 49 were successfully sequenced for both the 16S and 23S rRNA genes and the sequences subsequently assigned to 11 nucleotide sequence types (ntSTs). The 16S rDNA analysis revealed one *Mhf* group, at least three groups within *C*Mhm and at least two groups within *C*Mt. Notably, we identified *C*Mhm as well as two putative species closely related to *C*Mhm from 23S rDNA analysis, including one that has been previously identified. In contrast, the identity of the *C*Mt-derived 23S rDNA sequence ntST#11 remains unclear due to the lack of *C*Mt reference sequences, highlighting the need for more comprehensive *C*Mt data in public databases.

**Conclusions:**

Our data suggested the presence of two putative species related to *C*Mhm identified in domestic cats in Thailand. Integrating analyses of independent genetic markers, such as 16S and 23S rRNA genes, would enhance hemoplasma species identification and novel species discovery.

**Graphical abstract:**

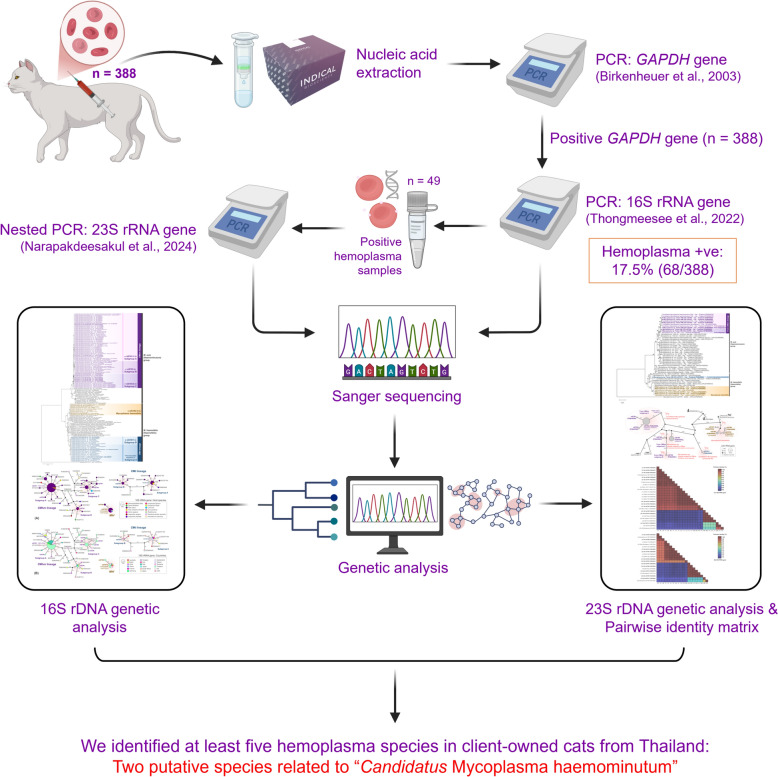

**Supplementary Information:**

The online version contains supplementary material available at 10.1186/s13071-025-07112-3.

## Background

Hemotropic *Mycoplasma* spp., commonly referred to as hemoplasmas, represent a distinct group of bacteria. These bacteria parasitize erythrocytes and induce infectious hemolytic anemia in a wide range of mammalian species, including cats [1–6]. *Mycoplasma haemofelis* (syn., *Haemobartonella felis*; *Mhf*), “*Candidatus* Mycoplasma (M.) haemominutum” (syn., “*Candidatus* Mycoplasma [M.] haematominutum”; *C*Mhm) and “*Candidatus* Mycoplasma (M.) turicensis” (syn., “*Candidatus* Mycoplasma [M.] turicense”; *C*Mt) are the three main *Mycoplasma* species infecting cats [1, 3, 5–7]. These three species have been documented in both domestic cats (*Felis catus*) [8–29] and wild felid species [30–37] in several countries worldwide, including Thailand.

Species identification and phylogenetic analyses of hemoplasmas are mainly based on 16S ribosomal DNA (rDNA) sequence characterization. Two hemoplasma groups can be delineated based on phylogeny: the Haemominutum group and the Haemofelis group [38–41]. Regarding feline hemoplasmas, *Mhf* and *C*Mt are placed in the Haemofelis group with *Mycoplasma haemocanis*, “*Candidatus* M. haemobos” and “*Candidatus* M. haemosuis,” while *C*Mhm is placed in the Haemominutum group with *Mycoplasma suis*, *Mycoplasma wenyonii*, *Mycoplasma ovis* and “*Candidatus* M. haematoparvum” [39, 40].

However, the 16S rRNA gene is unsuitable for characterizing hemoplasmas at the species level due to high sequence similarity [42]. For example, differentiating *M. suis* from *Mycoplasma parvum* in pigs using the 16S rRNA gene is challenging [40, 43, 44]. Therefore, other molecular markers (e.g. 23S rRNA, *dnaK*, *gyrB*, *rpoB* and *rpoC*) have been suggested to differentiate closely related hemoplasma species [42]. The use of both 16S and 23S rRNA gene targets was fundamental to uncovering species closely related to *M. suis* (a common hemoplasma species in pigs) and *M. wenyonii* (a common hemoplasma species in buffaloes and cattle) [39, 45, 46]. Similarly, using an integrated molecular approach for species identification, two *C*Mhm subgroups were identified and proposed to potentially contain novel feline hemoplasma species [41].

In the study reported here, we screened blood samples from a large cohort of cats using both 16S and 23S rRNA gene targets to advance current understanding of feline hemoplasma diversity and to identify potentially novel species within the *C*Mhm group. These hemoplasmas were further characterized through phylogenetic analyses and genetic network reconstruction.

## Methods

### Samples, data collection and nucleic acid extraction

In total, 388 blood samples from individually different cats were obtained from the Chulalongkorn University Veterinary Diagnostic Laboratory and the Veterinary Parasitology Unit (Chulalongkorn University, Bangkok, Thailand). All samples were submitted for laboratory testing (blood cytology or hematology) to the Small Animal Teaching Hospital, Faculty of Veterinary Science, Chulalongkorn University. For logistical convenience, laboratory staff supplied residual blood samples from healthy cats undergoing routine blood work and from sick cats. Information from individual cats (age, breed, neutering status, sex, and province) was retrieved from the Hospital Information System (HIS). Nucleic acid was extracted from each 200-µl blood sample using the IndiSpin® Pathogen Kit (Indical Bioscience GmbH, Leipzig, Germany) following the manufacturer’s instructions. All nucleic acid samples were stored at − 40 °C until further analysis.

### Successful nucleic acid extraction confirmation

Each nucleic acid sample was tested to confirm successful extraction using a PCR assay targeting the mammalian-endogenous glyceraldehyde-3-phosphate dehydrogenase gene (*GAPDH*) [47]. Each reaction volume consisted of a 12.5-µl PCR mixture containing 6.25 µl of 2X GoTaq® Green Master Mix (Promega Corporation, Madison, Wisconsin, USA), 3.5 µl of nuclease-free water, 0.375 µl of 10 µM forward primer (*GAPDH-F*), 0.375 µl of 10 µM reverse primer (*GAPDH-R*) and 2 µl of the sample. Pig genomic DNA and nuclease-free water were used as positive and negative controls, respectively. PCR tubes were placed into a T100™ Thermal Cycler (Bio-Rad Laboratories, Hercules, CA, USA) for amplification as follows: pre-denaturation at 94 °C for 3 min; 40 cycles of denaturation at 94 °C for 45 s, annealing at 49 °C for 45 s and extension at 72 °C for 1 min; and a final extension at 72 °C for 7 min (Additional file 1: Table S1). PCR products were electrophoresed for 35 min at 110 V and 400 mA in a 1.5% agarose gel (Bio Basic Inc., Markham, ON, Canada) mixed with RedSafe™ Nucleic Acid Staining Solution (iNtRON Biotechnology, Seongnam, Gyeonggi-do, South Korea). Expected bands (approx.  400 bp) were visualized using a UV transilluminator. Samples without a *GAPDH* band were excluded from further analysis.

### Screening for hemoplasma infection

Each sample was screened for hemoplasma infection using a PCR assay with primers targeting the 16S rRNA gene, as described in a previous study [40]. Each 25-µl PCR mixture contained 12.5 µl of 2X GoTaq® Green Master Mix, 7 µl of nuclease-free water, 0.75 µl of 10 µM forward primer (HM_16SF1), 0.75 µl of 10 µM reverse primer (HM_16SR1) and 4 µl of the sample. *C*Mhm-infected samples and nuclease-free water were used as positive and negative controls, respectively. PCR tubes were placed in a T100™ Thermal Cycler for amplification as follows: pre-denaturation at 94 °C for 3 min; 40 cycles of denaturation at 94 °C for 45 s, annealing at 55 °C for 45 s and extension at 72 °C for 1 min; and a final extension at 72 °C for 7 min (Additional file 1: Table S1). PCR products were electrophoresed, and expected bands (approx. 1,000 bp) were purified using the GenepHlow™ Gel/PCR Kit (Geneaid Biotech Ltd., Taipei, Taiwan) following the manufacturer’s protocol.

### Genetic analysis of the 16S rRNA gene

Each sample was sequenced bidirectionally (forward and reverse) by the U2Bio DNA Sequencing Service (U2Bio Co., Ltd., Bangkok, Thailand). All 16S rRNA sequences were evaluated and trimmed using MEGA X software [48]. The number of nucleotide sequence types (ntSTs) was analyzed using DnaSP 6.12.03 [49]. Percent identity of each ntST with GenBank® sequences was determined by the nucleotide Basic Local Alignment Search Tool (BLASTn) (https://blast.ncbi.nlm.nih.gov/Blast.cgi) [50]. All ntSTs were aligned with other deposited hemoplasma sequences and an outgroup (*Mycoplasma bovis* strain CQ-W70; accession number CP005933) using CLUSTAL W in MEGA X [48, 51]. The best-fit model for phylogenetic analysis was selected using ‘Find Best DNA/Protein Models (ML)’ in MEGA X. The 16S rRNA phylogenetic tree was constructed in MEGA X using the maximum likelihood (ML) method, Tamura-Nei (T93) model, a distinct gamma distribution (G), all sites for gaps/missing data treatment and 1000 bootstraps. Genetic networks based on the Templeton Crandall–Sing (TCS) method (host species and countries) were generated using PopART version 1.7 [52, 53]. Sequence Demarcation Tool version 1.3 (SDTv1.3) [54] was used to create a pairwise identity matrix of all ntSTs with selected GenBank® sequences.

### Genetic analysis of the 23S rRNA gene

Partial 23S rRNA sequences (approx. 1200 bp) were amplified from hemoplasma-infected samples using a nested PCR assay with primers from a previous study [38]. For the first round, each PCR mixture contained 12.5 µl of 2X GoTaq® Green Master Mix, 7 µl of nuclease-free water, 0.75 µl of 10 µM outer forward primer (Hm23SF1), 0.75 µl of 10 µM outer reverse primer (Hm23SR1) and 4 µl of the sample. PCR cycling conditions were: denaturation at 94 °C for 3 min; 35 cycles of denaturation at 94 °C for 45 s, annealing at 51 °C for 45 s and extension at 72 °C for 1 min 30 s); with a final extension at 72 °C for 7 min. The second round used the same reagent volumes with inner forward (Hm23SF2) and reverse (Hm23SR2) primers and 4 µl of the first-round product; cycling conditions were: denaturation at 94 °C for 3 min; 35 cycles of denaturation at 94 °C for 45 s, annealing at 50 °C for 45 s and extension at 72 °C for 1 min 30 s; with a final extension at 72 °C for 7 min (Additional file 1: Table S1). PCR products were electrophoresed, purified and sequenced as with the 16S rRNA gene.

All 23S rDNA sequences were analyzed similarly to 16S rDNA sequences, including ntST analysis, BLASTn, phylogeny, genetic network and pairwise identity matrix. The 23S rDNA phylogenetic tree was constructed using MEGA X with the ML method, T92 model, a distinct Gamma distribution (G), all sites for gaps/missing data treatment and 1000 bootstraps. Genetic networks were generated using PopART version 1.7 based on the TCS method, with host information.

###  feline leukemia virus/feline immunodeficiency virus testing

Frozen blood samples were thawed at room temperature for 15–20 min. The WITNESS® FeLV/FIV Rapid Test (Zoetis Inc., Parsippany, NJ, USA) was used to detect feline leukemia virus (FeLV) antigen and feline immunodeficiency virus (FIV) antibody in each sample, according to the manufacturer’s instructions. FeLV and FIV infection statuses were recorded for association analyses.

### Data analysis

The IBM SPSS Statistics Version 29.0 program (IBM Corp., Armonk, NY, USA) was used to calculate hemoplasma infection prevalence in cats with a 95% confidence interval (CI) using a one-sample nonparametric test. Associations between hemoplasma infection status and variables (age, breed, neutering status, sex, FeLV/FIV infection status and province) were evaluated using Pearson’s chi-square (*χ*^2^) test.* P*-values < 0.05 were considered to be statistically significant.

## Results

Blood samples from 388 cats were screened for hemoplasma infection using a PCR assay targeting the 16S rRNA gene [40], with the results showing that 68 cats (17.5%, 95% CI 13.9–21.7%) tested positive. Table 1 shows the infection status and its association with various variables. Hemoplasma was detected in all categories of each variable. Infection status was significantly associated with age (*χ*^2^ = 12.644), breed (*χ*^2^ = 25.082) and FIV infection status (*χ*^2^ = 23.762) (all *P* < 0.05). Hemoplasma infection was positively associated with age; prevalence rose across age categories. Non-pedigree status and FIV seropositivity were also associated with higher odds of infection.

**Table 1:** Association between hemoplasma infection status and cat-related variables

Information variables	Number of cats (%)			*χ*^2^	*P*-value
	Positive PCR for hemoplasma infection	Negative PCR for hemoplasma infection	Total		
*Age (n = 378)*				12.644	0.005*
≤ 1 year	1 (0.3)	34 (9.0)	35 (9.3)		
> 1 and ≤ 5 years	21 (5.6)	144 (38.1)	165 (43.7)		
> 5 and ≤ 10 years	23 (6.1)	76 (20.1)	99 (26.2)		
> 10 years	19 (5.0)	60 (15.9)	79 (20.9)		
*Breed (n = 388)*				25.082	< 0.001*
Pedigree	1 (0.3)	98 (25.3)	99 (25.5)		
Nonpedigree/DSH	67 (17.3)	222 (57.2)	289 (74.5)		
*Neutering status (n = 359)*				2.954	0.086
Neutered	45 (12.5)	166 (46.2)	211 (58.8)		
Not neutered	21 (5.8)	127 (35.4)	148 (41.2)		
*Sex (n = 379)*				1.142	0.285
Male	42 (11.1)	170 (44.8)	212 (55.9)		
Female	26 (6.9)	141 (37.2)	167 (44.1)		
*Province (n = 388)*				0.764	0.382
Bangkok	54 (13.9)	238 (61.3)	292 (75.3)		
Outside Bangkok	14 (3.6)	82 (21.1)	96 (24.7)		
*Status of FeLV infection (n = 388)*				3.462	0.063
Positive FeLV antigen	13 (3.4)	35 (9.0)	48 (12.4)		
Negative FeLV antigen	55 (14.2)	285 (73.5)	340 (87.6)		
*Status of FIV infection (n = 388)*				23.762	< 0.001*
Positive FIV antibody	13 (3.4)	11 (2.8)	24 (6.2)		
Negative FIV antibody	55 (14.2)	309 (79.6)	364 (93.8)		
*All cats (n = 388)*	68 (17.5, 95% CI 13.9–21.7)	320 (82.5, 95% CI 78.3–86.1)	388 (100)		

*Statistically significant association with hemoplasma infection status at *P* < 0.05

*CI* Confidence interval, *DSH* domestic short hair,* FeLV* feline leukemia virus,* FIV* feline immunodeficiency virus

Of the 68 hemoplasma-positive samples, 49 were successfully sequenced for both the 16S and 23S rRNA genes. Each gene was assigned to 11 ntSTs. Percent identity for each sequence was determined using BLASTn of the National Center for Biotechnology Information (NCBI). The BLASTn results on the 16S rDNA and 23S rDNA sequences are summarized in Tables 2 and 3, respectively. For the 16S rRNA gene, eight ntSTs (ntST#1–8) were closely aligned to *C*Mhm (DQ157144 [18], EU839985 [55], MN543623 [31], FJ004275 [56]); two ntSTs (ntST#9 and 10) were closely aligned to *Mhf* (KR905464); and one ntST (ntST#11) was closely aligned to *C*Mt (DQ464424) [57]. For the 23S rRNA gene, six ntSTs (ntST#1–6) were closely aligned to *C*Mhm (HE613254) [58]; four ntSTs (ntST#7–10) were closely aligned to *Mhf* (CP002808 [59, 60] and FR773153 [61]); and one ntST (ntST#11) was closely aligned to *Mycoplasma* sp. (OQ518944).

**Table 2:** Nucleotide sequence types (ntST) of 16S rDNA sequences from feline hemoplasmas analyzed by DnaSP6, nucleotide BLAST (BLASTn), phylogeny, and genetic network

ntST (GenBank® accession no.)	Number of samples collected (total collected = 49)	Blastn					Classified species in the phylogeny	Classified species in the genetic network
		Closest sequence	Species	Percent identity	Host	Country		
1 (PQ653823)	26	DQ157144	“*Candidatus* Mycoplasma haemominutum” 1020.17	100	Domestic cat	Switzerland	“*Candidatus* Mycoplasma haemominutum” group A	“*Candidatus* Mycoplasma haemominutum” group A
2 (PQ653824)	1	DQ157144	“*Candidatus* Mycoplasma haemominutum” 1020.17	99.90	Domestic cat	Switzerland	“*Candidatus* Mycoplasma haemominutum” group A	“*Candidatus* Mycoplasma haemominutum” group A
3 (PQ653825)	2	EU839985	“*Candidatus* Mycoplasma haemominutum” IT238_17	100	Domestic cat	Italy	“*Candidatus* Mycoplasma haemominutum” group B	“*Candidatus* Mycoplasma haemominutum” group B
4 (PQ653826)	2	MN543623	*Mycoplasma* sp. A1 (“*Candidatus* Mycoplasma haemominutum”)	99.90	Domestic cat	Chile	“*Candidatus* Mycoplasma haemominutum” group C	“*Candidatus* Mycoplasma haemominutum” group C
5 (PQ653827)	7	MN543623	*Mycoplasma* sp. A1 (“*Candidatus* Mycoplasma haemominutum”)	99.90	Domestic cat	Chile	“*Candidatus* Mycoplasma haemominutum” group C	“*Candidatus* Mycoplasma haemominutum” group C
6 (PQ653828)	1	MN543623	*Mycoplasma* sp. A1 (“*Candidatus* Mycoplasma haemominutum”)	99.79	Domestic cat	Chile	“*Candidatus* Mycoplasma haemominutum” group C	“*Candidatus* Mycoplasma haemominutum” group C
7 (PQ653829)	2	FJ004275	“*Candidatus* Mycoplasma haemominutum” Purdue	99.90	Domestic cat	USA	“*Candidatus* Mycoplasma haemominutum” group C	“*Candidatus* Mycoplasma haemominutum” group C
8 (PQ653830)	1	MN543623	*Mycoplasma* sp. A1 (“*Candidatus* Mycoplasma haemominutum”)	99.79	Domestic cat	Chile	“*Candidatus* Mycoplasma haemominutum” group C	“*Candidatus* Mycoplasma haemominutum” group C
9 (PQ653831)	4	KR905464	*Mycoplasma haemofelis* 57/10	99.89	Domestic cat	Italy	*Mycoplasma haemofelis*	*Mycoplasma haemofelis*
10 (PQ653832)	2	KR905464	*Mycoplasma haemofelis* 57/10	100	Domestic cat	Italy	*Mycoplasma haemofelis*	*Mycoplasma haemofelis*
11 (PQ653833)	1	DQ464424	“*Candidatus* Mycoplasma turicensis” D7	100	Domestic cat	South Africa	“*Candidatus* Mycoplasma turicensis” group B	“*Candidatus* Mycoplasma turicensis” group B

*BLASTn* Nucleotide Basic Local Alignment Search Tool,* ntST* nucleotide sequence type

**Table 3:** Nucleotide sequence types of 23S ribosomal DNA sequences from feline hemoplasmas analyzed by DnaSP6, nucleotide BLAST, phylogeny and genetic network

ntST (GenBank® accession no.)	Number of samples collected (total collected = 49)	Classification from the 16S rRNA gene	Blastn					Classified species in the phylogeny	Classification of species in the genetic network
			Closest sequence	Species	Percent identity	Host	Country		
1 (PQ645058)	1	“*Candidatus* Mycoplasma haemominutum” group A (16S-ntST#1 and 2)	HE613254	“*Candidatus* Mycoplasma haemominutum” Birmingham1	99.43	Domestic cat	UK	“*Candidatus* Mycoplasma haemominutum”	“*Candidatus* Mycoplasma haemominutum”
2 (PQ645059)	25		HE613254	“*Candidatus* Mycoplasma haemominutum” Birmingham1	99.53	Domestic cat	UK	“*Candidatus* Mycoplasma haemominutum”	“*Candidatus* Mycoplasma haemominutum”
3 (PQ645060)	1		HE613254	“*Candidatus* Mycoplasma haemominutum” Birmingham1	99.43	Domestic cat	UK	“*Candidatus* Mycoplasma haemominutum”	“*Candidatus* Mycoplasma haemominutum”
4 (PQ645061)	2	“*Candidatus* Mycoplasma haemominutum” group B (16S-ntST#3)	HE613254	“*Candidatus* Mycoplasma haemominutum” Birmingham1	95.65	Domestic cat	UK	A putative novel species	A putative novel species
5 (PQ645062)	12	“*Candidatus* Mycoplasma haemominutum” group C (16S-ntST#4‒8)	HE613254	“*Candidatus* Mycoplasma haemominutum” Birmingham1	96.69	Domestic cat	UK	Previously described novel putative species	Previously described novel putative species
6 (PQ645063)	1		HE613254	“*Candidatus* Mycoplasma haemominutum” Birmingham1	96.78	Domestic cat	UK	Previously described novel putative species	Previously described novel putative species
7 (PQ645064)	1	*Mycoplasma haemofelis* (16S-ntST#9 and 10)	CP002808	*Mycoplasma haemofelis* Ohio2	99.81	Domestic cat	USA	*Mycoplasma haemofelis*	*Mycoplasma haemofelis*
8 (PQ645065)	2		CP002808	*Mycoplasma haemofelis* Ohio2	100	Domestic cat	USA	*Mycoplasma haemofelis*	*Mycoplasma haemofelis*
9 (PQ645066)	2		FR773153	*Mycoplasma haemofelis* Langford1	99.90	Domestic cat	UK	*Mycoplasma haemofelis*	*Mycoplasma haemofelis*
10 (PQ645067)	1		FR773153	*Mycoplasma haemofelis* Langford1	100	Domestic cat	UK	*Mycoplasma haemofelis*	*Mycoplasma haemofelis*
11 (PQ645068)	1	“*Candidatus* Mycoplasma turicensis” group B (16S-ntST#11)	OQ518944	*Mycoplasma* sp. A2	91.98	Black mastiff bat	Belize	“*Candidatus* Mycoplasma turicensis”	“*Candidatus* Mycoplasma turicensis”

*BLASTn* Nucleotide Basic Local Alignment Search Tool,* ntST* nucleotide sequence type,* rRNA* ribosomal RNA

**Fig. 1:**
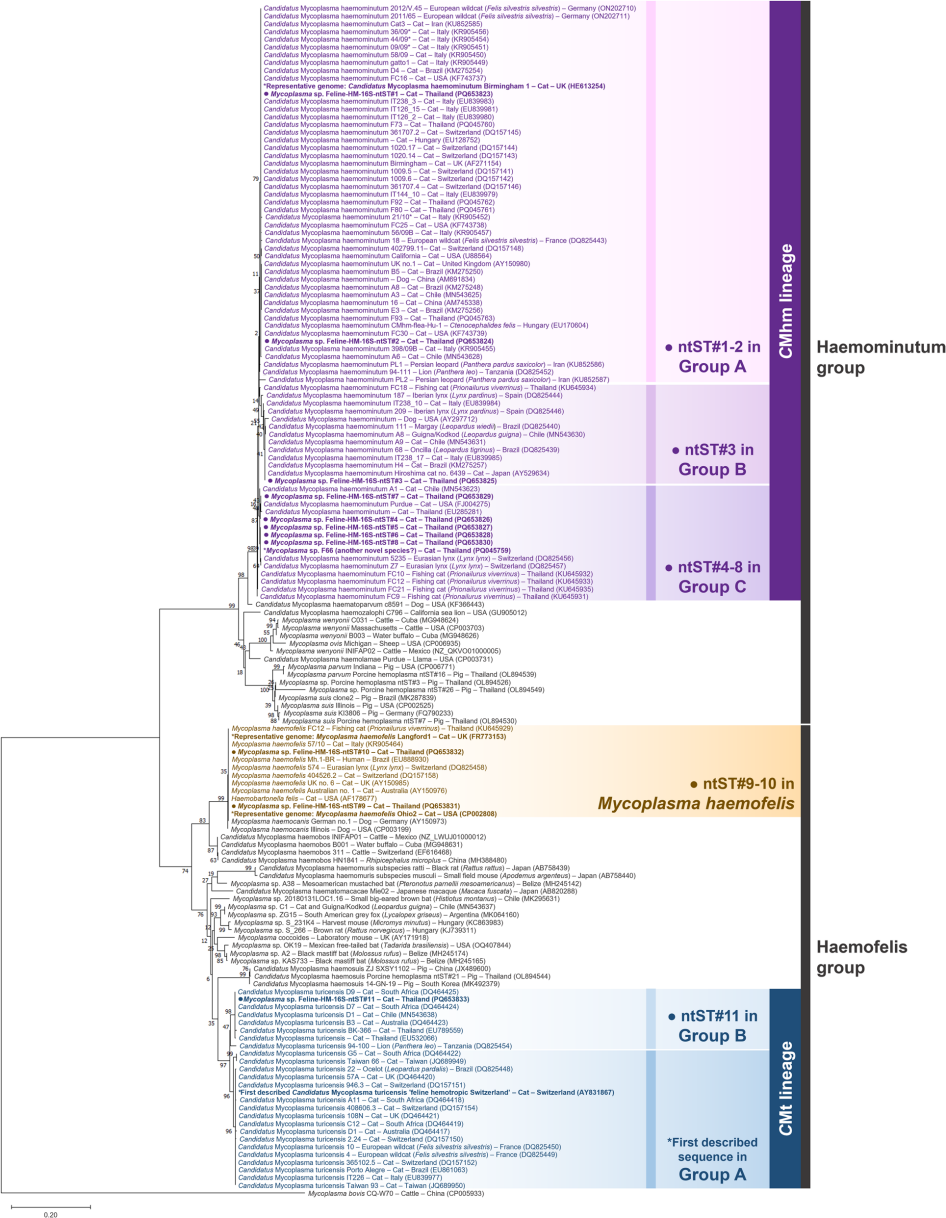
Phylogenetic relationships between the 16S rDNA sequences obtained in this study (filled circle) and other hemoplasma species deposited in GenBank® were analyzed. The tree was constructed using MEGA X software based on the maximum likelihood method, the Tamura-Nei (T93) model with a gamma distribution (G) and 1000 bootstraps. *Mycoplasma bovis* strain CQ-W70 (accession number CP005933) was used as the outgroup. An asterisk (*) indicates a previously identified representative genome or sequence used for comparison with the obtained sequences. *C*Mhm, “*Candidatus* Mycoplasma haemominutum” (syn., “*Candidatus* Mycoplasma haematominutum”); *C*Mt, “*Candidatus* Mycoplasma turicensis” (syn., “*Candidatus* Mycoplasma turicense)”; ntST, nucleotide sequence type; rDNA, Ribosomal DNA

**Fig. 2:**
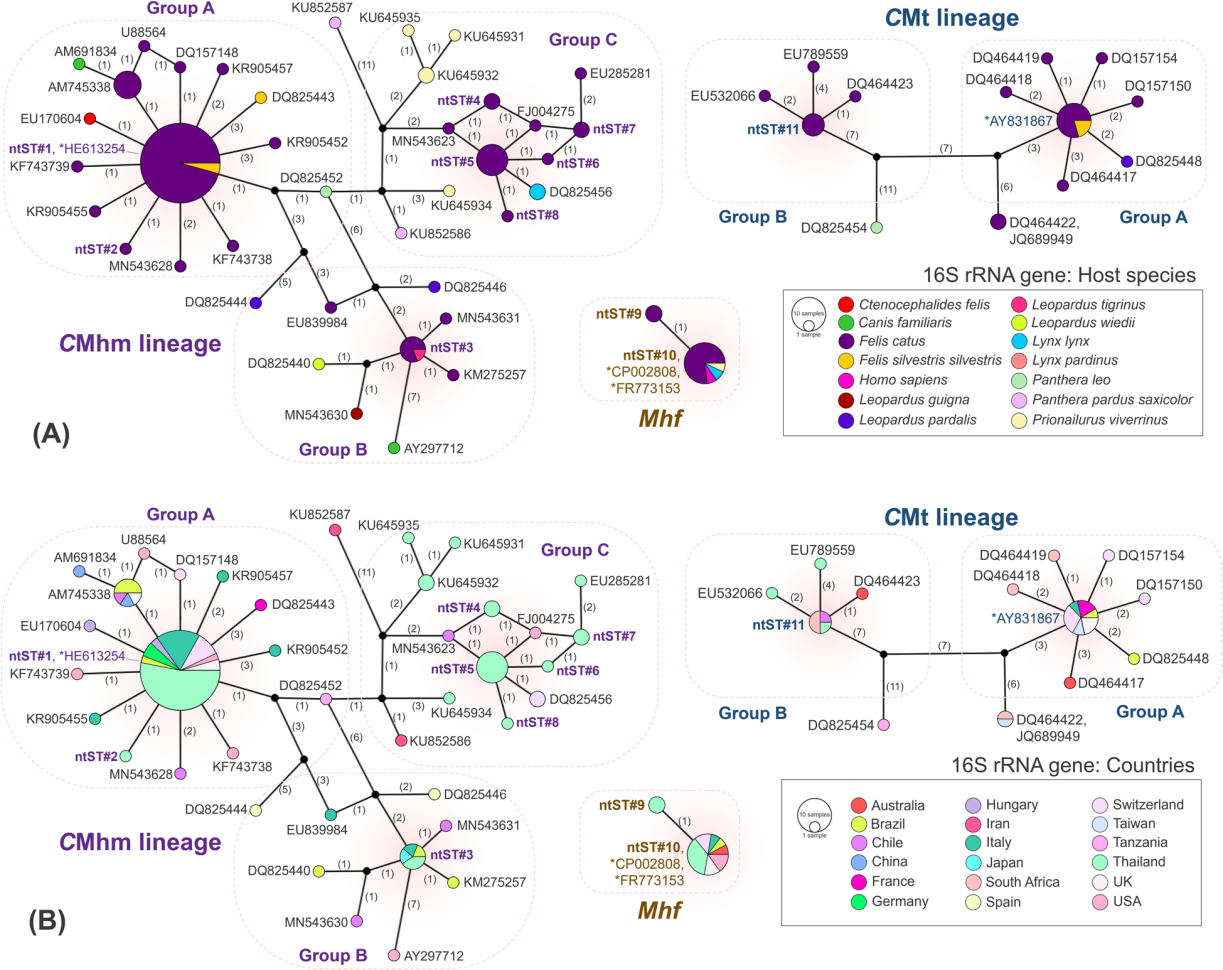
Genetic network of the 16S rDNA sequences and related hemoplasma sequences obtained in this study. The network was generated in PopART 1.7 using the Templeton Crandall–Sing method. Each circle represents a ntST. The color within each circle indicates the host species (**A**) or the country of origin (**B**). The number between two circles represents the number of nucleotide differences. The label next to each circle shows the GenBank® accession number corresponding to each ntST. An asterisk (*) indicates a previously identified representative genome or sequence. *C*Mhm, “*Candidatus* Mycoplasma haemominutum” (syn., “*Candidatus* Mycoplasma haematominutum”); *C*Mt, “*Candidatus* Mycoplasma turicensis” (syn., “*Candidatus* Mycoplasma turicense)”; *Mhf*, *Mycoplasma haemofelis* (syn., *Haemobartonella felis*); ntST, nucleotide sequence type; rDNA, ribosomal DNA

For the phylogenetic analysis of the 16S rRNA gene (Fig. 1), hemoplasma sequences were divided into the Haemominutum and Haemofelis groups, respectively. Sequences from this study (ntST#1‒11) clustered into three feline hemoplasma species: *Mhf*, *C*Mhm and *C*Mt. Specifically, ntST#1 to 8 belonged to the *C*Mhm lineage in the Haemominutum group; ntST#9 and 10 to the *Mhf* cluster in the Haemofelis group; and ntST#11 to the *C*Mt cluster in the same group. *C*Mhm sequences were further grouped into groups A (with HE613254, ntST#1 and ntST#2), B (with EU839985 and ntST#3) and C (with PQ045759, MN543623, FJ004275 and ntST#4 to 8). *C*Mt sequences were grouped into groups A (with AY831867) and B (with ntST#11). The TCS genetic network of the 16S rRNA gene (Fig. 2) also showed phylogenetic tree-like groupings based on host species and countries.

**Fig. 3:**
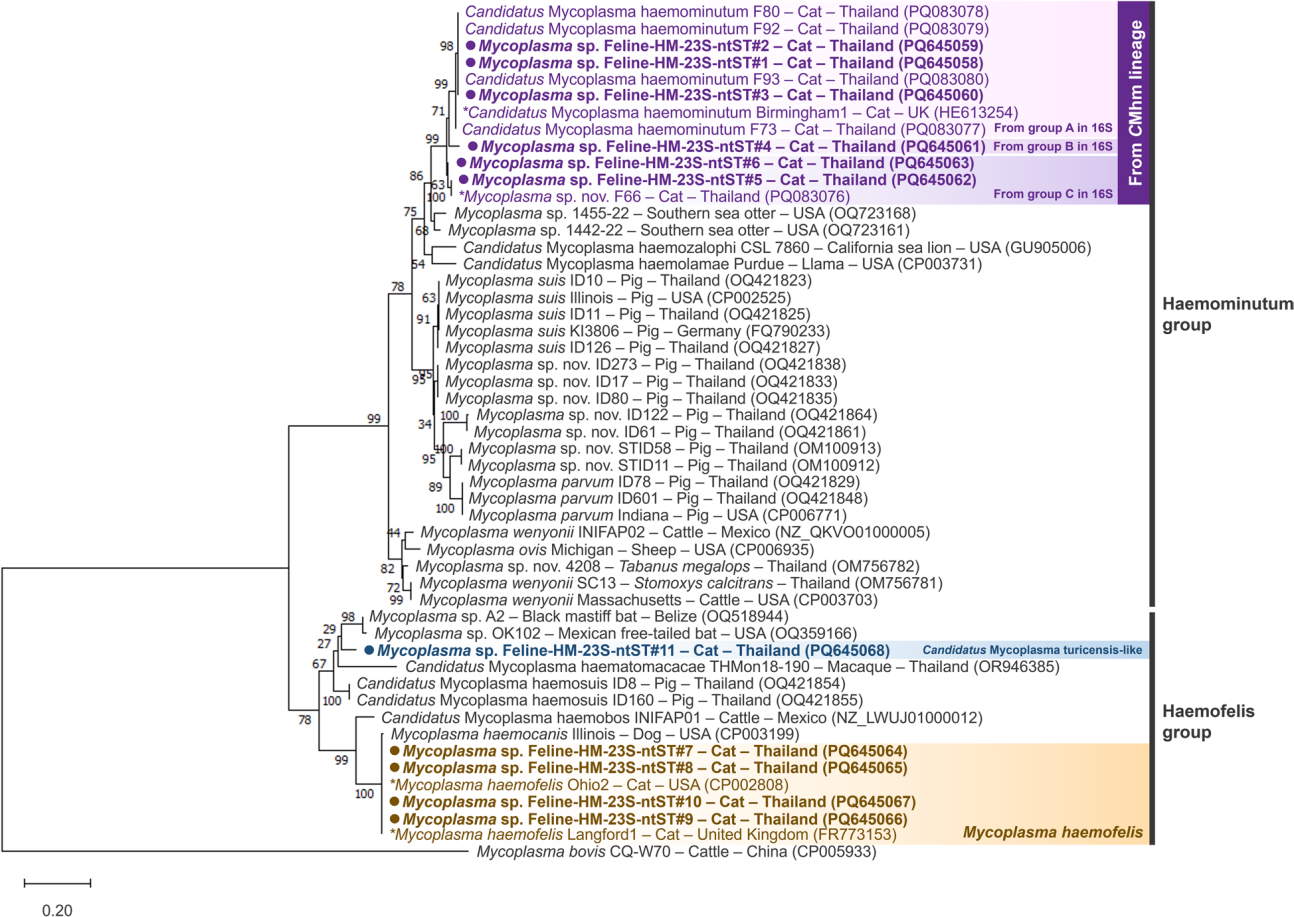
Phylogenetic relationships of the 23S rDNA sequences obtained in this study (filled circle) and their related hemoplasmas. The tree was constructed using MEGA X software based on the maximum likelihood method, the Tamura 3-parameter (T92) model with a gamma distribution (G) and 1000 bootstraps. *Mycoplasma bovis* strain CQ-W70 (accession number CP005933) was used as the outgroup. An asterisk (*) indicates a previously identified representative genome or sequence used for comparison with the obtained sequences. *C*Mhm, “*Candidatus* Mycoplasma haemominutum” (syn., “*Candidatus* Mycoplasma haematominutum”); ntST, nucleotide sequence type; rDNA, ribosomal DNA

**Fig. 4:**
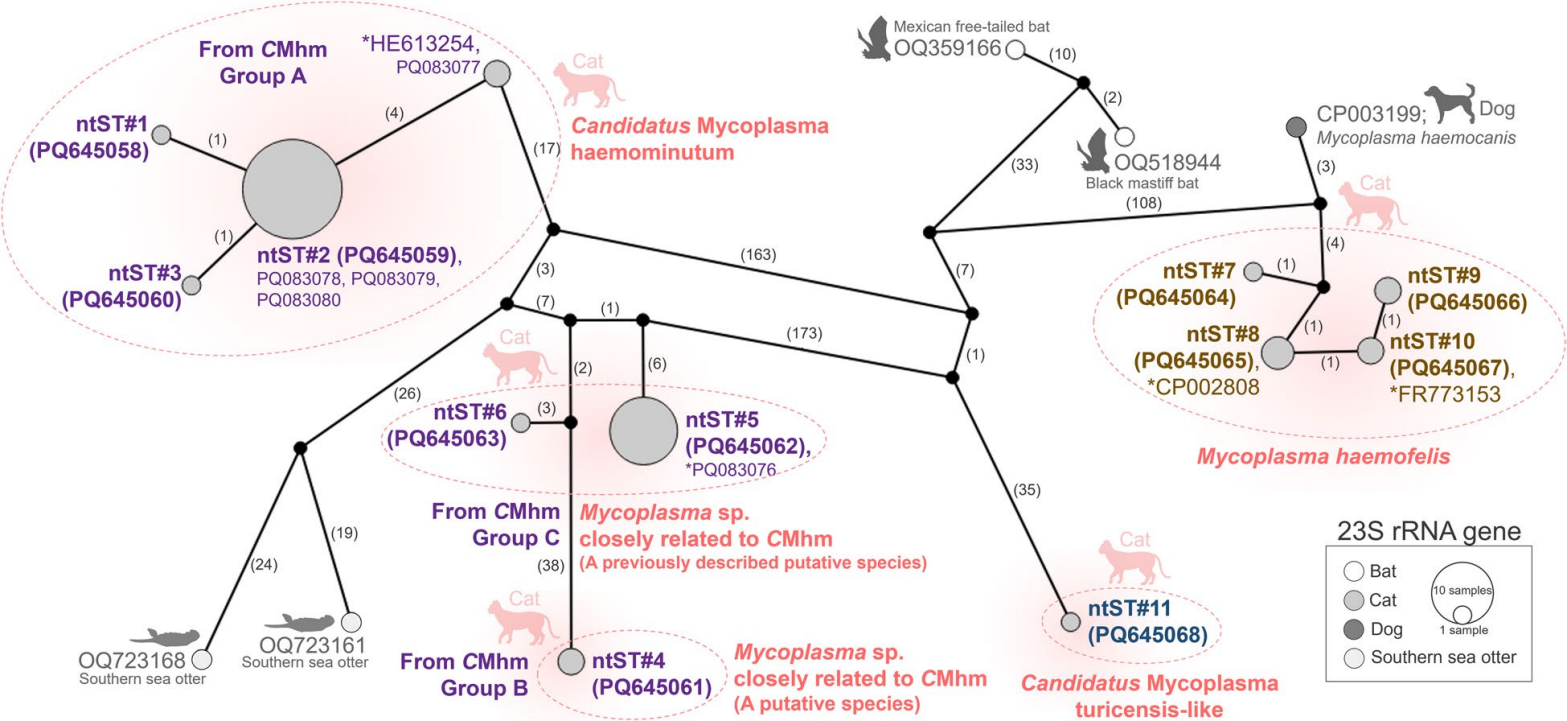
Genetic network of the 23S rDNA sequences and their related hemoplasmas obtained in this study. The network was generated in PopART 1.7 based on the Templeton Crandall–Sing method. Each circle and its color represent the ntST and host species, respectively. The number between two circles represents the number of nucleotide differences. The label next to each circle shows the GenBank® accession number representing each ntST. An asterisk (*) indicates a previously identified representative genome or sequence. *C*Mhm, “*Candidatus* Mycoplasma haemominutum” (syn., “*Candidatus* Mycoplasma haematominutum”); ntST, nucleotide sequence type; rDNA, ribosomal DNA

Hemoplasma sequences of the 23S rRNA gene were also divided into two groups in the phylogenetic tree (Fig. 3). Sequences from *Mhf* and *C*Mt were placed in the Haemofelis group, whereas sequences from *C*Mhm were placed in the Haemominutum group. Additionally, there were three groups most closely aligned to *C*Mhm in the 23S rRNA gene. The TCS genetic network of the 23S rRNA gene (Fig. 4) clearly demonstrated the genetic distances among species within these subgroups, which could not be resolved using 16S rDNA sequences alone. Results from the 23S rDNA phylogenetic tree and genetic network suggest that the “*C*Mhm lineage” samples (based on 16S rDNA) may represent at least three different species: *C*Mhm (group A; ntST#1 and 2), a previously described putative species (group C; ntST#5 and 6) and a putative species (group B; ntST#4). The identity of ntST#11 (PQ645068) remains unclear as to whether it represents true *C*Mt or a closely related species, due to the limited availability of *C*Mt 23S rDNA sequences in GenBank®.

**Fig. 5:**
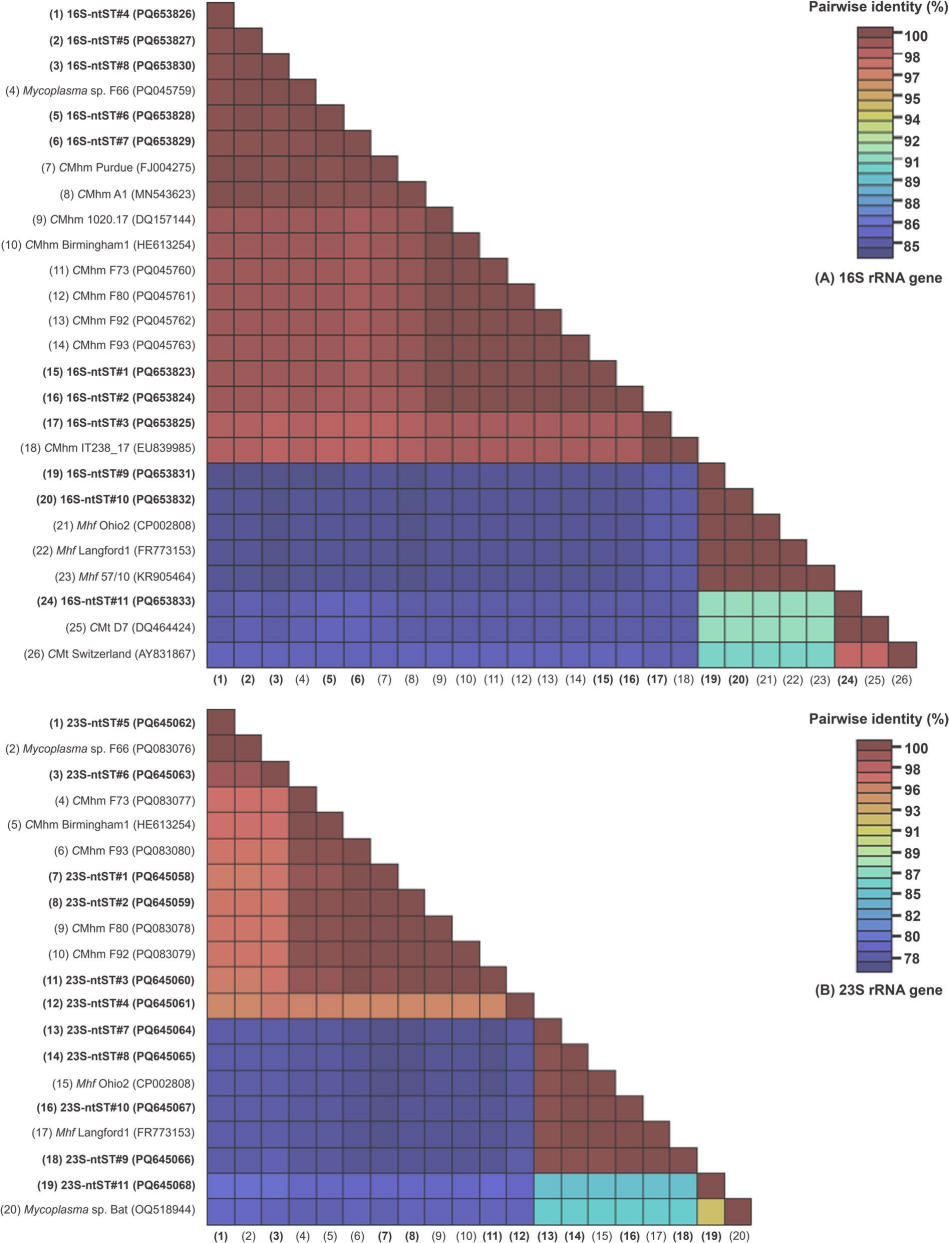
Pairwise identity matrix of 16S rDNA (**A**) and 23S rDNA (**B**) sequences obtained in this study and selected hemoplasma sequences from GenBank®. *C*Mhm, *Candidatus* Mycoplasma haemominutum” (syn., “*Candidatus* Mycoplasma haematominutum”); *C*Mt, “*Candidatus* Mycoplasma turicensis” (syn., “*Candidatus* Mycoplasma turicense)”; *Mhf*, *Mycoplasma haemofelis* (syn., *Haemobartonella felis* “); ntST, nucleotide sequence type; rDNA, ribosomal DNA

The *C*Mhm (23S-ntST#1 and 2) was the most abundant species (*n* = 27), followed by a previously described putative species (23S-ntST#5 and 6; *n* = 13), *Mhf* (23S-ntST#7–10; *n* = 6), a putative species (23S-ntST#4; *n* = 2) and *C*Mt-like sequences (23S-ntST#11; *n* = 1). Figure 5 presents the pairwise identity matrix of both 16S and 23S rRNA genes, showing percent identity among ntSTs and representative sequences.

## Discussion

This study provides the most comprehensive analysis of feline hemoplasmas in Thailand using both 16S and 23S rRNA genes and highlights the need for additional gene markers (e.g. 23S rRNA, *dnaK*, *gyrB*, *rpoB* and *rpoC*) beyond the 16S rRNA gene to elucidate feline hemoplasma genetic variability [42]. For example, while the 23S rRNA gene was used to characterize hemoplasmas at the species level in various mammals, including pigs [40, 45, 46], wildlife animals [38, 62–71] and hematophagous flies from buffalo farms [39], we recently showed that the 16S rRNA gene is too conserved to detect novel species within the *C*Mhm lineage [41].

In this study, using both rRNA gene analyses, we found that cats in Thailand are infected by at least five hemoplasma species, suggesting that some pathogens remain cryptic. Two of these species are abundantly reported in the literature, i.e., *Mhf* (16S-ntST#9 and 10; 23S-ntST#7–10) and the original *C*Mhm (16S-ntST#1 and 2; 23S-ntST#1–3). The latter includes all sequences grouped with the Birmingham 1 [58] or California [72–74] strains. Another species (16S-ntST#4–8; 23S-ntST#5 and 6) was linked to a previously described putative species (PQ083076) that is closely related to *C*Mhm [41]. We also characterized a putative novel species (16S-ntST#3; 23S-ntST#4) closely related to *C*Mhm, and another species (16S-ntST#11; 23S-ntST#11) reported as being *C*Mt-like. In Thailand, at least one of the three recognized feline hemoplasma species—*Mhf*, *C*Mhm and *C*Mt—has been detected in either domestic cats [19–21, 75–77], ectoparasites from cats [78] or wild felids such as fishing cats (*Prionailurus viverrinus*) [34, 77]. Different feline species may harbor diverse genotypes, as shown by the variability in genotypes reported in domestic and fishing cats [20]. In the present study, most 16S rDNA sequences from fishing cats grouped within *C*Mhm group C (16S-ntST#4‒8), possibly representing a putative species closely related to *C*Mhm [41]. Kaewmongkol et al. [20] also found a distinct genotype closely related to *Mhf* (MK632343) in cats using only the 16S rRNA gene. Thus, using the 23S rRNA gene and other markers would enhance identification and characterization of closely related hemoplasma species [42, 79–82]. For example, our sequences 16S-ntST#3 and 23S-ntST#4 are reported herein as a putative novel species; however, their 16S rDNA-like sequences have been reported as *C*Mhm in other studies [30, 31, 55, 83, 84], underscoring the need for 23S rRNA gene characterization to reveal cryptic hemoplasma diversity. Additionally, 16S-ntST#3 showed high nucleotide identity (99.48–100%) with several *C*Mhm sequences from domestic cats in Brazil (KM275257), Chile (MN543631) [31], Italy (EU839984 and EU839985) [55], Israel (AY150974) [84] and Japan (AY529634) [83], and also from wild felids in Brazil (DQ825439, DQ825440) [30], Chile (MN543630) [31] and Spain (DQ825445 and DQ825446) [30]. The 16S rRNA gene network for *C*Mhm group B suggests that this one putative novel species may occur in domestic cats and wild felids from several countries, including Brazil, Chile, Italy, Japan, Spain and Thailand, suggesting its potential global distribution, including Thailand.

In line with our findings, previous analyses of the *C*Mt 16S rRNA gene have divided this species into several sequence types or variants [24, 57], suggesting the existence of at least two groups. Recently, *C*Mt sequences from Egyptian cats were characterized into two clades [29]. In 2005, *C*Mt was first described in a domestic cat from Switzerland (AY831867) [85]. In phylogenetic analyses (Fig. 1), the first described *C*Mt sequence was placed in *C*Mt group A, while the sequence from this study (16S-ntST#11; PQ653833) was placed in *C*Mt group B. The genetic network developed in this study revealed more than two groups, with several nucleotide differences occurring in the *C*Mt lineage. For example, sequences from a cat in South Africa (DQ464422) [57] and from a cat in Taiwan (JQ689949) [26] showed at least nine (0.96%) and 20 (2.14%) nucleotide differences from ntST in groups A and B, respectively. A sequence from a lion (*Panthera leo*) in Tanzania (DQ825454) [30] showed at least 21 (2.14%) and 18 (1.93%) nucleotide differences from ntST in *C*Mt groups A and B, respectively. Collectively, the reported variability in the 16S rRNA gene among *C*Mt sequences across hosts and regions [39–41, 45] suggests that these groups and sequences (DQ464422, JQ689949 and DQ825454) could be closely related species. The sequence 16S-ntST#11 characterized in the present study could represent another putative novel species. However, the lack of *C*Mt 23S rDNA sequences from prior studies impedes comparison with our 23S-ntST#11 (PQ645068) and prevents further conclusions to be drawn on this isolate’s validity. Further 23S rRNA gene analysis with more *C*Mt-infected isolates is needed to confirm the presence of these novel species in Thailand and elsewhere.

We reported that 17.5% of client-owned cats harbor at least one hemoplasma species. These pathogens appear to be more prevalent in semidomesticated cats (38.05%) [19] and stray cats (46.0%) [21], suggesting lifestyle (e.g. roaming) is a risk factor. Moreover, older cats in our cohort had higher odds of testing positive, as previously reported in Thailand [19, 86]. FIV infection was also strongly associated with hemoplasma infection in this study, as in a previous study from Thailand [86]. Prior to this study, risk factors for hemoplasma infection were reported from Germany, with higher risks associated with male cats, outdoor access, multi-cat environment and FIV and FeLV infections [87] . However, in the present study, frozen (− 20 freezer storage) EDTA whole blood samples were thawed for 15–20 min to ambient temperature and used to study the status of retroviral infections. According to suggestions of the manufacturer of the test used to detect FeLV antigen and FIV antibody (Zoetis Inc.), “samples (serum and plasma only) should be kept frozen ( − 20 °C) for prolonged storage” (see topic IV: Sample storage; https://www.zoetis.es/_locale-assets/spc/witness-felv-fiv.pdf). Thus, our FeLV/FIV testing procedure was an extra-label use of the test.

Although the 23S rRNA gene has been widely used and claimed to identify hemoplasmas at the species level in various mammalian species [38, 40, 45, 46, 62, 71], the use of only two genetic markers (16S rRNA and 23S rRNA genes) to confirm a putative novel species of hemoplasma is sometimes overrated. Researchers should use the term “putative” or “potential” to establish an idea of being a novel hemoplasma species of those sequences. Moreover, an amplification of other genetic markers should be performed to ensure the species identified by the 23S rRNA gene are actually novel. For example, the RNase P RNA (*rnpB*) gene has also been used to characterize several hemoplasma species [40, 43, 88]. Due to limited resources and primer limitations (80F1/290R1) [89], this gene has not been amplified in the present study. Additional DNA sequencing and characterization of the *rnpB* gene or multi-gene sequencing (16S rRNA, 23S rRNA and *rnpB* genes) in a future study would enhance species characterization of hemoplasmas, especially in cats.

Severe disease following hemoplasma infection has been reported, necessitating species identification for clinical relevance. Multiplex PCR assays targeting the 16S rRNA gene have been developed to detect hemoplasma species in Thailand [77] and Turkey [90], but they are limited to *Mhf*, *C*Mhm and *C*Mt. In clinical settings, molecular diagnostics using universal primers (e.g. 16S rRNA gene) in anemic patients may offer a cost-effective diagnostic approach. The 16S rRNA gene-based PCR assay used in this study can also amplify other bacteria [39, 40]. In animals with signs of sepsis, using the 16S rRNA gene as a target should be done cautiously, given the presence of several pathogenic bacteria in mammals. Recently, a nanopore-based sequencing method accurately profiled the “hemobacteriome” from blood via full-length 16S rRNA gene sequencing [91]. While this approach may benefit large-scale epidemiological studies on hemobacteria diversity, its clinical utility remains limited.

Cats can also be infected with non-cat-specific species. *Mycoplasma wenyonii* and “*Candidatus* M. haemobos” were reported in cats from China [92] and Turkey [93]. Cats infected with “*Candidatus* M. haematoparvum”-like species were reported in Portugal [94] and the USA [95]. One European wild cat (*Felis silvestris*) in Niedersachsen, Germany was found to be infected with *M. ovis* [33], a common goat and sheep pathogen. Conversely, feline hemoplasmas have been reported in humans (*Mhf*-like) [96] and dogs (*C*Mhm) [97–100]. Thus, cross-species transmission may occur from other hosts to cats or vice versa. These findings highlight the importance of next-generation sequencing assays to better understand bacterial infections in mammalian blood, including those caused by hemoplasmas.

## Conclusions

In this study, the percentage of hemoplasma infection in client-owned cats from Thailand was 17.5% (68/388, 95% CI 13.9%–21.7%). At least five feline hemoplasma species were characterized in Thai client-owned cats, including *Mycoplasma haemofelis*, “*Candidatus* Mycoplasma haemominutum” (*C*Mhm), “*Candidatus* Mycoplasma turicensis”-like species and two putative novel species closely related to *C*Mhm, based on the 16S and 23S rRNA genes. We characterized two putative species related to *C*Mhm and demonstrated their natural circulation in cats in Thailand. Furthermore, we highlighted the importance of integrating analyses of independent genetic markers within the hemoplasma group for species discovery.

## Supplementary Information

Additional file 1: Table S1. Primer sequences and PCR conditions used in this study.

## Data Availability

All hemoplasma sequences obtained in this study can be found in GenBank®. For 16S rRNA sequences, ntST#1 to 11 were submitted under accession numbers PQ653823–PQ653833. For 23S rRNA sequences, ntST#1 to 11 were submitted under accession numbers PQ645058–PQ645068.
