# Supplementary material for: Hidden hemoplasma species within the “Candidatus Mycoplasma haemominutum” lineage in Thai cats revealed by analyses of two independent genetic markers
Source: Parasit Vectors. 2025 Nov 19;18:471. doi: 10.1186/s13071-025-07112-3 (PMC12629033; doi:10.1186/s13071-025-07112-3)
Supplement: Supplementary file 1 — Additional file 1: Table S1. Primer sequences and PCR conditions used in this study. [file 13071_2025_7112_MOESM1_ESM.docx]

**Supplementary Table 1.** Primer sequences and PCR conditions used in this study.

| Target gene | Primer name | Sequence (5′ to 3′) | Cycling conditions | Amplicon size (bp) | Reference |
| --- | --- | --- | --- | --- | --- |
| *GAPDH* | *GAPDH-F* | CCTTCATTGACCTCAACTACAT | Initial denaturation at 94°C for 3 min; 40 cycles of 94°C for 45 s, 49°C for 45 s, and 72°C for 1 min; a final extension at 72°C for 7 min; and a hold at 12°C | ∼450 | [47] |
|  | *GAPDH-R* | CCAAAGTTGTCATGGATGACC |  |  |  |
| 16S rRNA | HM_16SF1 | GAACAGCCGCAATGGGATTGAG | Initial denaturation at 94°C for 3 min; 40 cycles of 94°C for 45 s, 55°C for 45 s, and 72 °C for 1 min; final extension at 72°C for 7 min; hold at 12°C | ∼1,000 | [40] |
|  | HM_16SR1 | GACCTGGGAACGTATTCACCCTG |  |  |  |
| 23S rRNA | Outer Hm23SF1 | GGCTAGSGGTGAAATTCCAATCG | First round nPCR: 94°C for 3 min (initial denaturation); 35 cycles of 94°C for 45 s, 51°C for 45 s, and 72°C for 1 min 30 s; final extension at 72°C for 7 min; hold at 12°C | ∼1,200 | [38] |
|  | Outer Hm23SR1 | GTAAAGCTTCAYAGGGTCTTTCCGTC |  |  |  |
|  | Inner Hm23SF2 | GCCGAAATAGYTTTAGGACTAGCG | Second-round nPCR: 94°C for 3 min (initial denaturation); 35 cycles of 94°C for 45 s, 50°C for 45 s, and 72°C for 1 min 30 s; final extension at 72°C for 7 min; hold at 12°C |  |  |
|  | Inner Hm23SR2 | CTGCAGYCGAGACAGTTAAGRG |  |  |  |

All PCR reactions were performed using GoTaq® Green Master Mix (Promega, USA) as the main reagent.
